# Serum BAFF levels, Methypredsinolone therapy, Epstein-Barr Virus and Mycobacterium avium subsp. paratuberculosis infection in Multiple Sclerosis patients

**DOI:** 10.1038/srep29268

**Published:** 2016-07-07

**Authors:** Giuseppe Mameli, Eleonora Cocco, Jessica Frau, Giannina Arru, Elisa Caggiu, Maria Giovanna Marrosu, Leonardo A. Sechi

**Affiliations:** 1 Dipartimento di Scienze Biomediche, Sezione di Microbiologia e Virologia, Università di Sassari, Italy; 2 Centro Sclerosi Multipla, Dipartimento di Sanità Pubblica Medicina Clinica e Molecolare, Università di Cagliari, Via Is Guadazzonis 2, 09126 Cagliari, Italy

## Abstract

Elevated B lymphocyte activating factor BAFF levels have been reported in multiple sclerosis (MS) patients; moreover, disease-modifying treatments (DMT) have shown to influence blood BAFF levels in MS patients, although the significance of these changes is still controversial. In addition, BAFF levels were reported increased during infectious diseases. In our study, we wanted to investigate on the serum BAFF concentrations correlated to the antibody response against *Mycobacterium avium* subspecies *paratuberculosis* (MAP), Epstein-Barr virus (EBV) and their human homologous epitopes in MS and in patients affected with other neurological diseases (OND), divided in Inflammatory Neurological Diseases (IND), Non Inflammatory Neurological Diseases (NIND) and Undetermined Neurological Diseases (UND), in comparison to healthy controls (HCs). Our results confirmed a statistically significant high BAFF levels in MS and IND patients in comparison to HCs but not NIND and UND patients. Interestingly, BAFF levels were inversely proportional to antibodies level against EBV and MAP peptides and the BAFF levels significantly decreased in MS patients after methylprednisolone therapy. These results implicate that lower circulating BAFF concentrations were present in MS patients with humoral response against MAP and EBV. In conclusion MS patients with no IgGs against EBV and MAP may support the hypothesis that elevated blood BAFF levels could be associated with a more stable disease.

Multiple sclerosis (MS) is an autoimmune central nervous system disease where T cells play a central role in disease pathogenesis. Recently B cells and antibodies are increasingly recognized as important elements in the pathogenesis of MS and are target in different trials^1^,^2^. It has been reported an intrathecal antibody (Ab) production and B-cell expansion in MS lesions^3^. However, no specific MS biomarkers have been validated for clinical use, including specific antibodies as immunological markers of MS^4^.

B-cell activating factor (BAFF), a member of the tumor necrosis factor family, is the major survival factor for B cells^5^. It has an essential role in B-cell homeostasis and in the development of several autoimmune diseases, (i.e. systemic lupus erythematosus, rheumatoid arthritis, primary Sjögren’s, myasthenia gravis, systemic sclerosis, Graves’ disease), furthermore BAFF blood levels were higher during different infectious diseases and its role in the maintenance of inflammation has been recognized^1^,^6^,^7^,^8^,^9^,^10^. Data concerning the BAFF serum circulating levels in MS patients are controversial and a significant difference in MS compared to healthy controls (HCs) has not been always demonstrated^11^,^12^,^13^. For this reason we wanted to investigate the correlation of serum BAFF levels and antibodies titer against selected peptides derived from Epstein-Barr Virus and *Mycobacterium avium* subspecies *paratuberculosis* previously associated with MS^14^,^15^,^16^,^17^,^18^,^19^. B cell activation leads to proliferation and Ab production that can protect from pathogens or promote autoimmunity^2^,^3^. Moreover, only few studies investigated the influence of MS treatments and blood BAFF levels.

Krumbholz *et al*.^9^ reported comparable BAFF serum concentrations in untreated MS patients and HCs, and the treatment with (IFN)-β brings to an up-regulation of BAFF. Others studies reported that treatment with IFN-β was associated with higher serum BAFF levels^6^.

Short-term Methylprednisolone treatment (1000 mg for 3–5 days) improves MS symptoms during relapses but does not stop disease progression^13^.

A recent study investigated blood BAFF levels after a short course of Methylprednisolone treatment in MS patients, and not significantly variation of BAFF levels in 65% of relapsing-remitting MS patients was reported^11^.

Some authors report that BAFF levels are lower in subjects with high specific IgE to *Ascaris*, suggesting that this cytokine plays a role in the strength of the Ab responses to this nematode [10].

The purpose of this study was to determine whether BAFF levels: (i) were increased in MS patients in comparison to other neurological diseases (OND) and HC, (ii) were modified after Methylprednisolone therapy in MS patients (iii) were correlated to the humoral response against peptides from infectious agents, previously associated to MS: Epstein-Barr Virus (EBV) and *Mycobacterium avium* subsp. *paratuberculosis* (MAP)^14^,^17^,^18^,^19^.

## Results

**Figure 1:**
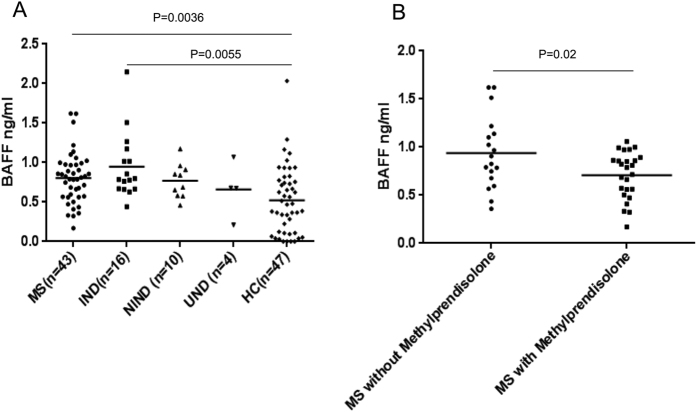
Levels of serum BAFF among MS, IND, NIND, UND and HCs. (**A**) BAFF levels were statistically significant higher in MS and IND compared with the HCs (MS versus HC, p = 0.0002; OND versus HC, p = 0.0002). (**B**) BAFF levels within subgroups of MS patients, the level of BAFF was lower in Methylprednisolone treated MS patients than untreated MS patients (p = 0.02).

**Figure 2:**
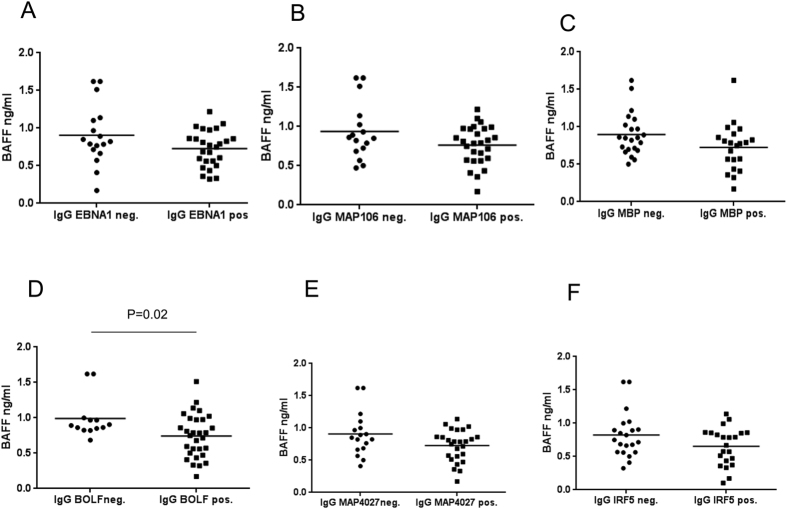
Figure shows BAFF serum levels in MS samples positive or negative to Abs against EBV, MAP and human homologous peptides, respectively: (**A**) EBV latent EBNA1_400–413_; (**B**) MAP_106c_121–132_ and (**C**) MBP_85–98_ and the other homologous peptides group; (**D**) EBV lytic BOLF1_305–320_; (**E**) MAP_4027_18–32_ and (**F**) IRF5_424–434_.P values were calculated by T Student test, Graph Pad Prism 6.0 software (San Diego, CA, USA).

**Figure 3:**
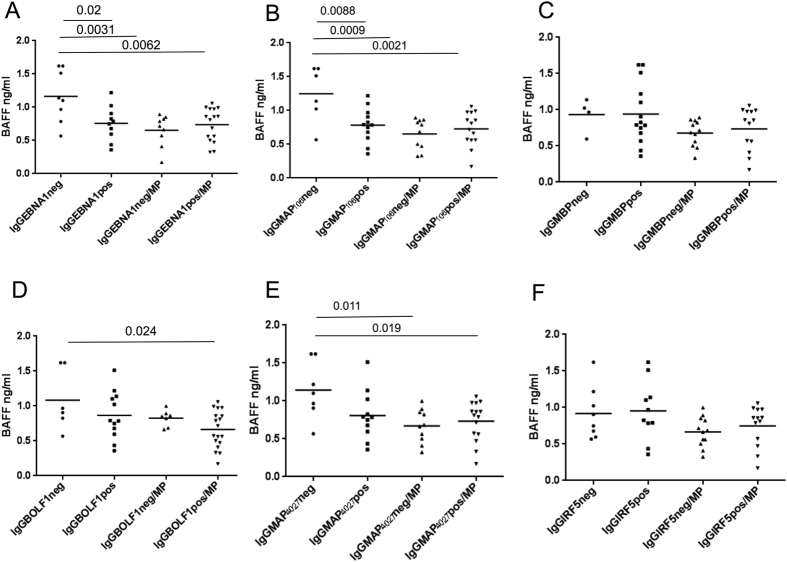
BAFF serum levels in MS samples with or without Methylprednisolone (MP) therapy positive or negative to EBV, MAP and human homologous peptides: EBV latent EBNA1_400–413_; (**A**) MAP_106c_121–132_; (**B**) MBP_85–98_. (**C**) EBV lytic BOLF1_305–320_; (**D**) MAP_4027_18–32_; (**E**) IRF5_424– 434_. (**F**) P values were calculated by Dunn’s test, Graph Pad Prism 6.0 software (San Diego, CA, USA).

We compared the concentration of circulating BAFF in plasma samples collected from MS, OND and HCs subjects. BAFF serum levels were significantly higher in MS patients (0.82 ng/ml Kruskal-Wallis test with Dunn’s multiple comparison test, p = 0.0036), IND patients (0.95 ng/ml; Kruskal-Wallis test with Dunn’s multiple comparison test, p = 0.0055) than in HCs (0.56 ng/ml) whereas BAFF levels were not statistically significant in NIND (0.77 ng/m) and UND patients (0.66 ng/ml) with Dunn’s multiple comparison test (Fig. 1A). Then we investigated if Methylprednisolone treatment in MS could influence the circulating BAFF levels. Results in Fig. 1B show that patients treated with Methylprednisolone had significant lower amounts of BAFF protein (0.63 ng/l; Student’s *t*-test p = 0.02) compared to patients without therapy (0.95 ng/ml). This explains also the lower levels of BAFF observed in MS patients with relapses (all treated with Methylprednisolone, data not showed). Since BAFF has been implicated in the strength of the antibody responses against to different infections^8^,^10^ and MAP and EBV have been associated to MS^14^,^15^,^16^,^17^,^18^,^19^, we investigated on the correlation between the humoral response against MAP and EBV specific epitopes, their human homologous peptides (MBP and IRF5) and plasma BAFF concentration. We found that BAFF protein levels were higher in MS patients with negative to EBNA1_400–413_, MAP_0106c_121–132_, MAP_4027_18–32_ and human MBP_85–98_, IRF5_424–434_ homologous peptides, but this data was not statistically significant. BAFF serum levels were lower in EBV and MAP positive MS patients in comparison to patients negative to MAP and EBV (Fig. 2A–F) at a statistic borderline level of significance, whereas BAFF levels were statistically significant higher in BOLF negative patients than in IgG BOLF1_305–320_ positive patients (p = 0.02, Fig. 2D). Moreover, we investigated MS patients divided according to Methylprednisolone (MP) therapy and according to IgG positivity or IgG negativity against EBV, MAP and human homologous peptides (Fig. 3). A statistically significant association between the level of serum BAFF protein in MS patients negative to EBNA1_400–413_ and BAFF serum levels in MS patients positive to: EBNA1 (Dunn’s multiple comparison test, p = 0.02, Fig. 3A), EBNA1 negative and positive under MP treatment (Dunn’s multiple comparison test, p = 0.0031 and p = 0.0062, respectively; Fig. 3A) was observed. The same results were obtained when MS patients MAP0106c_121–132_ negative were tested (Fig. 3B) where an higher BAFF serum level was found in MS patients MAP negative in comparison to MS patients MAP0106c_121–132_ positive (p = 0.0088), MS patients MAP0106c_121–132_ positive and negative under MP treatment (p = 0.0009 and p = 0.0021 respectively; Fig. 3B). No statistical significance in BAFF serum level was observed in MS patients negative and positive to the MBP homologous peptide, with or without MP therapy (Fig. 3C).

A BAFF higher serum concentration was found in MS patients negative to BOLF1 compared to MS patients positive to BOLF1 under MP treatment (Dunn’s multiple comparison test, p = 0.024, Fig. 3D).

A statistically significant association between the level of serum BAFF protein in MS patients negative to MAP_4027_18–32_ and BAFF levels in MS patients with MP therapy, negative and positive to MAP_4027_18–32_ was also observed (Dunn’s multiple comparison test p = 0.011 and p = 0.019 respectively; Fig. 3E).

Finally, no correlation was observed when we compared BAFF serum levels among MS patients negative and positive to the homologous peptide IRF5, with or without MP treatment (Fig. 3F).

## Discussion

MS has been considered a T cell-mediated disease, but new studies are reconsidering the importance of B cell-mediated immunity in disease progression^1^,^2^. BAFF is a B cell survival factor and a member of the TNF ligand superfamily, but it can also regulate T cells function^3^,^5^,^20^. It has been reported that the number of B cells and the presence of BAFF-binding receptors determine the concentrations of soluble BAFF^12^. BAFF is upregulated following bacterial, viral and protozoa exposure^7^,^8^,^21^ and inversely regulated to the antibody response against *Ascaris lumbricoides*^10^. BAFF is also upregulated in MS^3^, however, to date human studies have failed to establish a link between BAFF levels in serum or plasma and MS severity or progression linked to infection triggers^7^,^10^,^11^. In the present study, we confirmed that peripheral blood BAFF levels are significantly higher in MS and in IND patients compared to HC, but not in NIND and UND patients as expected^11^,^22^. Somewhat surprisingly, we observed a lower level of BAFF in Methylprednisolone treated patients, not observed in previous studies^11^,^22^. Furthermore, BAFF levels were significantly lower in relapsing remitting patients (data not showed), although this was probably due to the Methylprednisolone therapy (all of them were under treatment). We also tried to correlate circulating BAFF levels to antibody positivity against MAP and EBV infections previously found to be associated with MS^14^,^15^,^16^,^17^,^18^,^19^.In previous studies, a stronger humoral response against two EBV peptides EBNA1_400–413_ and BOLF1_305–320_, the homologous mycobacterial peptides MAP_0106c_121–132_ and MAP_4027_18–32_ and human homologues MBP_85–98_ and IRF5_424–434_ was detected in MS patients compared to HCs^14^,^15^,^16^,^17^,^18^,^19^. In contrast to what is generally observed in autoimmune diseases, we found an inverse association between soluble BAFF and the antibody response against EBV, MAP and the human homologous peptides. Indeed we observed an inverse correlation between BAFF levels and IgG positivity against EBNA1_400–413_, MAP_0106c_121–132_, and BOLF1_305–320,_ MAP_4027_18–32_ peptides in MS patients with and without Methylprednisolone therapy. Finally, we were not able to observe any statistically significant differences of BAFF peripheral blood level in MS patients positive or negative to the human homologous peptides MBP and IRF5 with or without Methylprednisolone therapy.

It has been reported that short-term treatment with high doses of intravenous methylprednisolone did not significantly alter plasma BAFF levels in 65% of relapsing-remitting MS patients^11^. Our results are partially in contrast to this observation, but are in line with a other report where stable MS patients without relapses exhibited significantly higher BAFF levels than relapsing patients and treatment with interferon-β and immunosuppressants raised BAFF blood levels^11^. The fact that MS patients negative to MAP and EBV have an higher peripheral BAFF concentration, suggest the hypothesis that elevated blood BAFF levels could be associated with a better disease outcome.

Finally, our study raise more questions on the role of BAFF in MS and its possible correlation to humoral-immune response against MAP and EBV, for this reason further studies are foreseen.

## Materials and Methods

### Subjects

MS samples were collected from 43 patients that fulfilled the revised McDonald diagnostic criteria (Table S1)^4^. Serum samples were collected from MS and Other Neurological Disease (OND) composed by Inflammatory Neurological Diseases (IND), Non Inflammatory Neurological Diseases (NIND) and Undetermined Neurological Diseases (UND). Serum samples from OND were collected from 30 patients (14 females and 16 males; mean age ± SD was 47 ± 24.6 years 16 IND (5 females and 11 males, mean age ± SD was 44 ± 25.6 years), 10 NIND (5 females and 5 males, mean age ± SD was 45 ± 22.1 years) and 4 UND (1 females and 3 males, mean age ± SD was 65 ± 112.8 years).

Samples from 47 HC matched with MS and OND patients (F/M = 23/24; mean age 38.0 ± 7.5) were collected at the Transfusion center of the Sassari Hospital. The study protocol was approved by the ethic committee of the University of Cagliari, Italy. All subjects approved the “informed consent” to the study. Methods were carried out in “accordance” with the approved guidelines. All the participants provided written consent. All samples were prospectively collected for diagnosis purposes and measured under equal conditions.

### BAFF ELISA

Serum samples obtained from MS, OND and HC patients were stored under −80 °C until the experiments were performed. Soluble BAFF levels were determined by quantitative BAFF Human ELISA KITs, (Abcam, England). The assay was performed in duplicate according to the manufacturers instructions. Absorbance was read at 450 nm with an automated 96-well plate reader (Spectramax Plus, Molecular Devices, Sunnyvale, CA, USA). Soluble BAFF concentrations were determined by interpolation with the standard curve and calculated as ng⁄ml.

### EBV, MAP and human homologues peptides

Synthetic peptides derived from EBV antigens (BOLF1_305–320_, EBNA1_400–413_), MAP homologues antigens (MAP_4027_18–32_, MAP_0106C_121–132_) and human homologues (MBP_85–98_, IRF5_424–434_) were included in the study; peptides were synthesized commercially (LifeTein, South Plainfield, NJ 07080 USA) with a purity >90% and kept frozen in single-use aliquots [10 mM] at −80 °C.

Indirect Enzyme-Linked Immunosorbent Assays (ELISA) was carried out to detect specific Abs for all the synthetic peptides (assayed at 10 μg/ml) included in the study as previously reported^14^.

### Statistical analysis

The analysis was performed using Graphpad Prism 6.0 software. The non-parametric Student’s *t*-test was as well used to compare the BAFF levels between two different groups. When more than two groups were analyzed, Kruskal-Wallis non parametric test and Dunn’s multiple comparison test were performed. A value of p < 0.05 was considered significant.

## Additional Information

**How to cite this article**: Mameli, G. *et al*. Serum BAFF levels, Methypredsinolone therapy, Epstein-Barr Virus and Mycobacterium avium subsp. paratuberculosis infection in Multiple Sclerosis patients. *Sci. Rep.* **6**, 29268; doi: 10.1038/srep29268 (2016).

## Supplementary Material

Supplementary Information
