# Supplementary material for: Serum BAFF levels, Methypredsinolone therapy, Epstein-Barr Virus and Mycobacterium avium subsp. paratuberculosis infection in Multiple Sclerosis patients
Source: Sci Rep. 2016 Jul 7;6:29268. doi: 10.1038/srep29268 (PMC4935889; doi:10.1038/srep29268)
Supplement: Supplementary Information [file srep29268-s1.pdf]

Serum BAFF levels, Methypredsinolone therapy, Epstein Barr Virus and Mycobacterium avium subsp. paratuberculosis infection in Multiple Sclerosis patients

Giuseppe Mameli,[1] Eleonora Cocco,[2] Jessica Frau,[2] Giannina Arru,[1] Elisa Caggiu,[1] Maria Giovanna Marrosu[2] and Leonardo A. Sechi[1*]

Table S1: MS patients details and therapy treatments.

Patients	Onset Disease	Blood samples	EDSS	Disease form	Relapse	MP therapy	IFN-beta therapy	Rituximab therapy
MS#1	1994	10/02/12	1	RR	YES	NO	NO	NO
MS#2	2003	05/09/14	1	RR	NO	NO	NO	NO
MS#3	2012	18/09/13	1	RR	YES	NO	NO	NO
MS#4	2012	11/05/13	1.5	RR	NO	YES	NO	NO
MS#5	1995	04/11/11	7	SP	YES	YES	NO	NO
MS#6	2007	15/05/12	2	RR	NO	NO	NO	NO
MS#7	2012	25/10/12	3	RR	NO	NO	NO	NO
MS#8	2011	04/10/11	0	RR	NO	YES	NO	NO
MS#9	2013	15/04/14	1	RR	YES	YES	NO	NO
MS#10	2012	09/05/14	1	RR	NO	NO	NO	NO
MS#11	2011	28/04/14	1	RR	NO	NO	NO	NO
MS#12	2011	22/10/13	1	RR	NO	NO	NO	NO
MS#13	2010	11/12/13	1	RR	NO	NO	NO	NO
MS#14	1993	17/05/12	6	SP	NO	NO	NO	NO
MS#15	2014	20/07/12	1	RR	YES	YES	NO	NO
MS#16	2012	08/02/12	3	RR	YES	YES	NO	NO
MS#17	2013	06/11/12	0	RR	YES	YES	NO	NO

MS#18	2012	14/09/11	0	RR	NO	NO	NO	NO
MS#19	2011	05/12/12	1.5	RR	YES	YES	NO	NO
MS#20	2013	14/06/12	2.5	RR	NO	NO	NO	NO
MS#21	2013	14/06/11	1.5	RR	YES	YES	NO	NO
MS#22	2012	03/12/13	1	RR	NO	NO	NO	NO
MS#23	2004	23/01/14	3	SP	NO	YES	YES	NO
MS#24	2008	12/04/12	6	SP	NO	YES	NO	NO
MS#25	2001	05/12/13	1.5	RR	YES	NO	NO	NO
MS#26	2011	07/03/14	2.5	RR	YES	YES	NO	NO
MS#27	2002	13/07/12	4.5	RR	NO	NO	NO	NO
MS#28	2010	29/06/12	2	RR	YES	YES	NO	NO
MS#29	2014	01/10/14	2	RR	YES	NO	NO	NO
MS#30	2012	04/09/13	1.5	RR	YES	YES	NO	NO
MS#31	2011	18/06/12	1	RR	YES	YES	NO	NO
MS#32	2012	29/11/12	1	RR	YES	YES	NO	NO
MS#33	2007	18/06/12	3.5	RR	YES	YES	NO	NO
MS#34	2013	08/05/14	3.5	RR	YES	YES	NO	NO
MS#35	2001	06/12/13	2	RR	YES	YES	NO	NO
MS#36	2013	06/09/12	3.5	RR	YES	YES	NO	NO
MS#37	2011	12/11/13	1.5	RR	YES	YES	NO	NO
MS#38	2008	16/04/14	3	RR	YES	YES	NO	NO
MS#39	2013	19/08/14	0	RR	YES	YES	NO	NO
MS#40	2012	06/10/11	1.5	RR	YES	YES	NO	NO
MS#41	2013	29/10/13	6	RR	NO	YES	NO	NO
MS#42	2013	25/03/14	3.5	RR	NO	NO	NO	NO
MS#43	2007	08/07/14	1.5	RR	NO	NO	NO	NO
